# Recent Advances in Nanomaterial-Based Chemiluminescence Probes for Biosensing and Imaging of Reactive Oxygen Species

**DOI:** 10.3390/nano13111726

**Published:** 2023-05-25

**Authors:** Chuanlin Huang, Wenjuan Zhou, Riliga Wu, Weijiang Guan, Nengsheng Ye

**Affiliations:** 1 Department of Chemistry, Capital Normal University, Beijing 100048, China; 2 State Key Laboratory of Chemical Resource Engineering, Beijing University of Chemical Technology, Beijing 100029, China

**Keywords:** reactive oxygen species, chemiluminescence, nanomaterial, biosensing, imaging

## Abstract

Reactive oxygen species (ROS) play important roles in organisms and are closely related to various physiological and pathological processes. Due to the short lifetime and easy transformation of ROS, the determination of ROS content in biosystem has always been a challenging task. Chemiluminescence (CL) analysis has been widely used in the detection of ROS due to its advantages of high sensitivity, good selectivity and no background signal, among which nanomaterial-related CL probes are rapidly developing. In this review, the roles of nanomaterials in CL systems are summarized, mainly including their roles as catalysts, emitters, and carriers. The nanomaterial-based CL probes for biosensing and bioimaging of ROS developed in the past five years are reviewed. We expect that this review will provide guidance for the design and development of nanomaterial-based CL probes and facilitate the wider application of CL analysis in ROS sensing and imaging in biological systems.

## 1. Introduction

Reactive oxygen species (ROS) are molecules or compounds with oxygen, hydrogen or nitrogen atoms that are more reactive than molecular triplet oxygen. They include nonradical ROS such as hydrogen peroxide (H_2_O_2_), singlet oxygen (O_2_), hypochlorous acid/hypochlorite (HClO/ClO^−^) and peroxynitrite (ONOO^−^), as well as free radicals such as hydroxyl radicals (•OH), superoxide (O_2_^•−^), nitric oxide (NO•) and alkoxyl radicals (RO•) [1,2]. Human physiological activities depend heavily on the equilibrium of the intracellular redox state, yet unbalanced metabolism causes oxidative stress [3,4]. ROS are primarily created during mitochondrial aerobic respiration and are regarded as a class of essential species in cell regulation, which are a significant class of oxidative species that play a role in a variety of physiological and pathological processes, including molecular signaling, neurological damage, inflammation, Alzheimer’s disease and cancer [5]. ROS can remove biological factors that are detrimental to the cell and serve a crucial part in cell signaling [6,7]. However, an excessive ROS level may result in extensive oxidative damage to different biomolecules, including proteins, DNAs and lipids, which causes several kinds of diseases and aging, such as neurological damage, inflammation, Alzheimer’s disease or cancer [8,9].

In the past dozen years, methods for ROS detection have proliferated significantly, including electron spin resonance (ESR), fluorescence (FL), spectrophotometry and phosphorescence [10,11,12,13]. Typically, in these procedures, a capture probe reacts with ROS to produce stable molecules that can potentially be detected in a further spectroscopic detection phase. This makes it extremely difficult for them to rapidly monitor ROS generation and transformation because they are labor-intensive, time-consuming, and often have low sensitivity in the presence of intrinsic background signals brought on by outside light sources [14]. Chemiluminescence (CL) is a method for ultrasensitive chemical analysis that relies on energy transfer from chemical reactions to excite specific molecules in the system and produce photons. Since the initial observation of horseradish peroxidase, also known as HRP, catalyzing the oxidation of luminol by H_2_O_2_, CL has developed swiftly because of the significant signal amplification. A variety of CL reagents have been identified and deployed, including luminol derivatives, lucigenin, peroxyoxalate, 1,2-dioxetane and its derivatives [15]. Due to the fact that CL is typically produced by oxidation reactions, it has the benefit of being rapid and suitable for short-lived ROS. Additionally, because there is no laser excitation, CL exhibits a high signal-to-noise ratio, making it one of the most sensitive detection techniques for ROS monitoring [13,14,15,16,17,18]. Even so, many of the current CL systems are inadequate for imaging ROS in complicated biological materials or living cells. For instance, the deep imaging of ROS in organs cannot be achieved with conventional luminol CL methods due to a maximum emission wavelength of 425 nm. Moreover, ROS are constantly developing and emerging in sophisticated biological systems, making the procedure of identifying ROS more challenging. A specific detection or highly sensitive detection of ROS is also a great challenge. Therefore, it is crucial to explore the construction of reliable CL probes for ROS analysis.

At present, nanomaterials are currently one of the most desirable material candidates for the majority of study fields based on their high specific surface area, high charge transfer, great optical characteristics, controlled functionalization and superior biocompatibility [19,20]. As nanotechnology has developed quickly, the introduction of nanomaterials in CL systems opens up new avenues for the creation of extremely effective CL systems. In the creation of CL sensors, a number of nanomaterials have attracted significant interest, including metal nanoparticles [21], graphene oxide [22], nanoclusters [23,24], quantum dots [25,26], carbon dots [27], layered double hydroxides [28] and metal–organic frameworks [29]. In this article, we reviewed the current status of CL in ROS biosensing and imaging and the application of nanomaterials in this context. The review was divided into two main parts; the first section described the different roles of nanomaterials in CL systems, for example, as catalysts, emitters and carriers of CL reagents. We separately discussed the applications of different categories of nanomaterials in CL systems according to their different roles. The second section was devoted to discussing in detail the application of nanomaterial-based CL probes for ROS bioassay in recent years. Nanomaterial-based CL probes for ROS (H_2_O_2_, •OH, O_2_^•−^, ^1^O_2_, ONOO^−^, HClO/ClO^−^) are reviewed separately. Finally, the prospects for the adoption of nanomaterials in the development of ideal CL probes for ROS were discussed, as well as the problems faced. This review will serve to direct the development of effective CL probes for ROS and expand the potential applications of nanomaterials for biosensing and imaging of ROS.

## 2. The Role of Nanomaterials in the CL System

Due to their large specific surface area and surface energy, quantum size effect, etc., nanomaterials present many special properties, such as catalytic activity, adsorption or loading capacity and electronic and optical properties [30,31]. Recently, the use of nanomaterials in CL systems for better analytical performance and create adaptable and stable CL probes has drawn a lot of interest [32,33]. Nanomaterials have been reported as catalysts, emitters, energy acceptors, and carriers for CL reagents or enzymes, resulting in high-performance CL platforms [34].

### 2.1. As Sensitizers and Catalysts

In various CL systems, a variety of nanomaterials have served as sensitizers and catalysts. In the last few years, the development of the luminol or its derivative L012-H_2_O_2_ CL platform has been facilitated by the exploitation of numerous nanomaterials, such as noble metal nanoparticles (NPs) and nanoclusters (NCs) [35], metal–organic frameworks (MOFs) [36,37], carbon nanomaterials [38], layered double hydroxides (LDHs) [39] and polymer dots (Pdots) [40].

**Figure 1:**
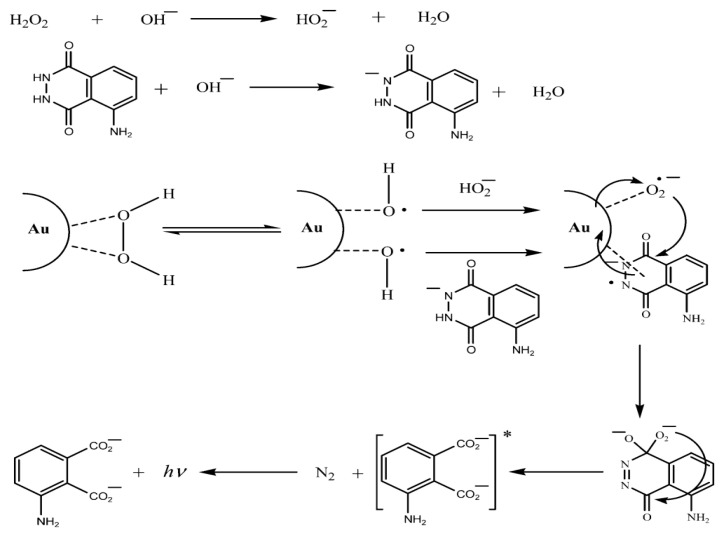
Possible mechanism for the Au-NP-enhanced CL of a luminol–H_2_O_2_ system. Reprinted with permission from ref. [41]. Copyright 2005 American Chemical Society.

As one kind of classical nanomaterial, NPs (e.g., metal, metal oxide or semiconductor NPs) show significant catalytic effects on certain CL reactions, especially in some H_2_O_2_-related CL systems, owing to their peroxidase-like activity that can initiate the transformation of H_2_O_2_ to •OH, thereby enhancing the CL signal (Figure 1) [41]. The most prevalent and typical of these are noble metal NPs, which include silver, gold and platinum [42]. Since 2005, Au NPs were the first to be reported for enhancing the CL signal of the luminol–H_2_O_2_ system [41], and increasing amounts of noble-metal-related nanomaterials have been exploited to create highly sensitive CL sensing platforms [43,44]. In recent years, noble metal NCs (such as AuNCs and AgNCs) have also been discovered to catalyze CL reactions in the luminol–H_2_O_2_ system with the development of fluorescent metal NCs [35,45]. Their facile production, adjustable fluorescence emission, and low toxicity make them an attractive candidate for use in catalytic CL in biological systems. Sheng et al. have shown that the luminol–H_2_O_2_ CL system exhibited obviously enhanced CL under catalysis of bovine serum albumin (BSA)-capped AgNCs [35].

**Figure 2:**
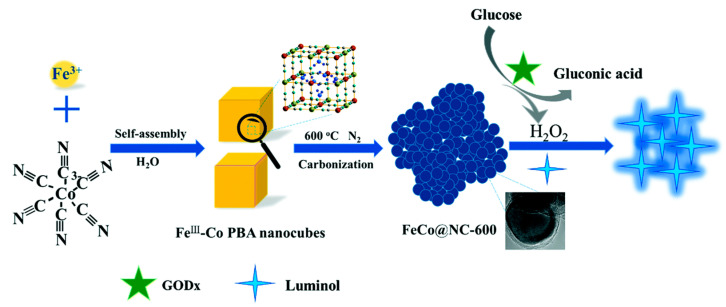
The creation of the FeCo@NC hybrids and their application as a peroxidase mimic for CL sensing of glucose. Reprinted with permission from ref. [48]. Copyright 2019 Royal Society of Chemistry.

Compared with single-metal NPs, bimetallic or alloy metal NPs integrate different metal elements into one NP system. Because of the synergistic catalysis of multiple metals, alloy nanoparticles often show better catalytic performance [46]. Au/Ag bimetallic alloy NPs were initially utilized for CL enhancement. In contrast to Au NPs, Au/Ag alloy NPs shown better catalytic properties for the luminol–H_2_O_2_ CL system. Additionally, Au/Cu and Fe/Co alloy nanoparticles have recently been utilized to boost the CL signals of luminol–H_2_O_2_ and rhodamine B-H_2_O_2_ systems [47,48]. By direct carbonization of the Fe^Ⅲ^-Co Prussian blue analogue, graphitic layers encapsulating FeCo alloy NPs (called FeCo@NC) were synthesized by Lu’s group (Figure 2) [48]. Due to the N-doping and unique structure, the FeCo@NC hybrids exhibited excellent CL catalytic activity, which enhanced the luminol–H_2_O_2_ CL emission intensity by more than 85-fold. High precision and repeatability have been achieved in the detection of hyperglycemia in human serum samples with the FeCo@NC-hybrid-based nanozyme. 

Similar to metal NPs, metal oxide NPs are frequently employed to catalyze the CL reaction of luminol–H_2_O_2_ because they have the capacity to accelerate the breakdown of H_2_O_2_ to create •OH. Chen et al. looked into the catalytic capabilities of CuO NPs for the luminol–H_2_O_2_ CL system for the first time and observed a considerable amplification of the CL signal [42]. Li et al. investigated the catalytic activity of α-Fe_2_O_3_ nanorods, cubiform Co_3_O_4_ NPs and NiO NPs on the CL reaction that occurs in the luminol–H_2_O_2_ system [49]. For the luminol system, all three types of NPs demonstrated high catalytic activity. The CL techniques based on the employing of Co_3_O_4_ nanoparticles showed particular sensitivity and specific selectivity.

Moreover, the porous metal–organic framework (MOF), which is a supramolecular coordination polymer with highly ordered porosity, large surface areas, variable pore sizes and superior stability, is greatly beneficial for improving catalysis and analysis performance in CL [50]. Inspired by the strategy of modifying MOFs with functionalized modulators, Mao’s group constructed the first β-CD (β-cyclodextrin) hybrids, which allowed the luminol–H_2_O_2_ system’s CL intensity to be increased 30-fold [36]. The remarkable catalytic activity of the MOF-235 frameworks is a consequence of their huge surface area and several open metal sites. The catalytic and CL mechanism is described below. In the luminol–H_2_O_2_ system, MOF-235/β-CD catalyzes the breakdown of H_2_O_2_ to generate active ROS intermediates (e.g., •OH), which then react with luminol to produce unstable excited 3-aminophthalate anions (3-APA*), which finally produce enhanced CL. The ability of β-CD to stabilize 3-APA also leads to enhanced CL. Tang et al. developed Fe_3_O_4_ and MOF MIL-101(Fe) composites that also exhibited excellent catalytic properties for the luminol–H_2_O_2_ CL system [37]. Zhu et al. developed a copper-based MOF called Cu-BTC (HKUST-1), which was tested for its catalytic influence on the CL reaction of the luminol–H_2_O_2_ system. It was discovered that, in alkaline medium, the CL intensity was significantly increased by 90-fold [51]. According to research, the catalytic mechanism of Cu-BTC (HKUST-1) involves the surface of the catalyst facilitating electron transport and radical production activities. Furthermore, a quick approach for detecting dopamine (DA) in biological samples was created based on DA’s inhibitory effect on the luminol–H_2_O_2_-HKUST-1 system. In 2021, Yang et al. employed a Cu metal–organic framework (Cu-MOF) to catalyze the luminol–H_2_O_2_ system, achieving persistent CL [52]. The continued emission of the luminol was probably due to the gradual decomposition of H_2_O_2_ to ^1^O_2_, •OH and O_2_^•−^ in the luminol–H_2_O_2_ system catalyzed by Cu-MOFs. The vast surface area of Cu-MOFs also allowed •OH and O_2_^•−^ to recombine into the comparably more durable ^1^O_2_ on its surface, prolonging the CL even more. These studies have provided new insights into the development of CL nanomaterial catalysts.

Carbon nanomaterials, graphene oxide (GO) and its derivatives have attractive catalytic potential due to their high adsorption capacity, huge specific surface area and biocompatibility. For example, Wang et al. observed that GO greatly enhanced ^1^O_2_-induced CL in the luminol–H_2_O_2_ system [38]. The catalytic effect of GO might be related to its effective catalysis of the breakdown of H_2_O_2_ and to the acceleration of electron transfer, producing a high yield of ^1^O_2_ on its surface. The ^1^O_2_-induced CL intensity of luminol could be enhanced 6-fold under GO catalysis. This work demonstrated new applications of GO in CL and contributed to the enrichment of the luminol CL mechanism. In addition, Liu et al. prepared N-aminobutyl-N-ethylisoluminol (ABEI) and horseradish peroxidase (HRR) bifunctionalized graphene oxide hybrids with a convenient strategy. These hybrids, called ABEI-GO@HRP, have shown outstanding CL activity when interacting with H_2_O_2_ under neutral and alkaline circumstances [53]. The intense CL emission may be explained by the fact that GO promoted the formation of •OH, O_2_^•−^ and −CO_4_^•2−^ in this CL reaction; simultaneously, •OH might add to double bonds at the GO plane to generate strongly oxidizing π-C=C^•^, which could react with ABEI in alkaline conditions. Furthermore, GO could serve as a reaction platform to facilitate electron transfer in reactions involving free radicals, thus enhancing the CL emission.

LDHs, with highly ordered structures, have caused a lot of fascination in the catalysis sector. In 2017, Pan et al. exploited LDH to enhance the CL of the H_2_O_2_ and luminol system [54]. The positively charged brucite-like layers of LDH and the intercalated carbonate, which have the ability to adsorb luminol dianions and peroxide anions and advertise the generation of carbonate radicals that promote the formation of the light-emitting intermediate 3-aminophthalate anions, were suggested as the mechanism of CL enhancement. Recently, Cheng et al. prepared an ionic liquid–LDH assembly via forming hydrogen-bonding interactions between the hydroxyl group on the surface of the LDH and the amino groups in 1-(3-aminopropyl)-3-methyl-imidazolium tetrafluoroborate([apmim]BF_4_) [55]. When a structurally organized [apmim]BF_4_ ionic liquid–LDH assembly was present, it was found that the CL signal of the luminol–H_2_O_2_ system was greatly increased. The increased production of •OH and O_2_^•−^ via rapid mass transport from the solution to the surface of the ionic liquid–LDH assembly was the cause of the amplified signal. These findings explored new opportunities for the development of structurally ordered catalysts to enhance CL emission. In addition to their excellent catalytic performance, LDHs are also considered as promising catalyst carriers. For instance, LDHs have been used to support MOF catalyst materials to prepare composite nanomaterials because of the unsaturated coordination state of its surface cations [39]. The produced LDH-based ZIF-8 nanocomposite LDH@ZIF-8 had high peroxidase-like activity for the breakdown of H_2_O_2_ into •OH, hence boosting the CL signal of the luminol–H_2_O_2_ system. 

Some functionalized polymer nanoparticles are also frequently used to catalyze CL reactions of a luminol–H_2_O_2_ system. Pdots are polymer nanoparticles formed by high amounts of polymer or small molecules moderately crosslinked or carbonized. The Pdots are also commonly used to support catalytic active materials due to their good water solubility and easy modification of surface functional groups. Excellent catalytic activity is shown by metalloporphyrins and hemi-functionalized Pdots in the luminol derivative L012-H_2_O_2_ system [40,56,57]. The strong and long-term CL emission makes it possible for ultrasensitive imaging of ROS in biological systems. 

The exploitation of these nanomaterials has attracted widespread research interest and has led to an increasing number of applications in CL catalysis. Nanocatalysts improve the intrinsic luminescence intensity and luminescence time of different CL systems, enhancing the sensitivity of detection and expanding their applications.

### 2.2. As Emitters or Energy Acceptor

Various fluorescent nanomaterials such as semiconductor quantum dots (QDs), carbon dots (CDs), nanocomposites and metal nanoclusters (MNCs) have been explored as luminophores or energy receptors in CL reactions, greatly enhancing the scope of CL applications and analytical detection capabilities. 

**Figure 3:**
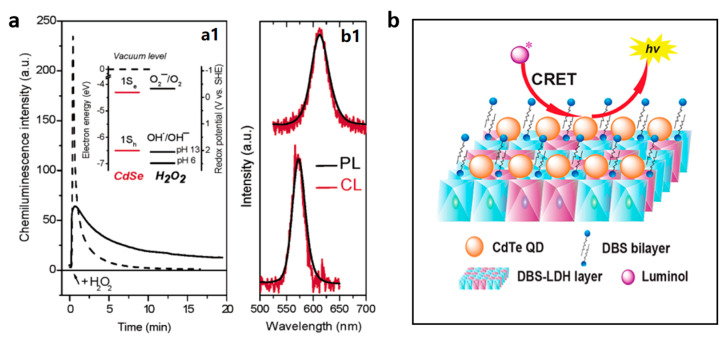
(**a**) CL of a CdSe/CdS nanocrystal film produced in an alkaline environment with the addition of H_2_O_2_. Reprinted with permission from ref. [58]. Copyright 2004 American Chemical Society. (**b**) QD-LDH nanocomposites for the enhancement of luminol–H_2_O_2_ CL. Reprinted with permission from ref. [62]. Copyright 2013 American Chemical Society.

Direct CL of QDs was first reported by Weller’s group in 2004 [58]. They prepared CdSe/CdS quantum dot films on Pt and F-doped SnO_2_ substrates. It is observed that CdSe/CdS QDs can emit light which shows the same wavelength as the fluorescence of CdSe/CdS QDs during the redox reaction with H_2_O_2_ under alkaline conditions. They hypothesized the mechanism of the QDs CL as follows: First, H_2_O_2_ is catalytically decomposed on the Pt and F-doped SnO_2_ substrates to produce •OH radicals (M + H_2_O_2_ → M^+^ + OH^−^ + •OH); Then, •OH injects holes into CdSe/CdS QDs to generate CdSe(h^+^_1S *h*_)/CdS (•OH + CdSe/CdS → OH^−^ + CdSe(h^+^_1S *h*_)/CdS); Meanwhile, •OH reacts with H_2_O_2_ to produce O_2_^•−^ under alkaline condition (•OH + H_2_O_2_ + OH^−^ → O_2_^•−^ 2H_2_O), which injects electrons into CdSe/CdS QDs to produce CdSe(^-^_1S *e*_)/CdS (O_2_^•−^ + CdSe/CdS →CdSe(^-^_1S *e*_)/CdS+ O_2_); The 1S_e_-1S_h_ transition emission gives rise to the CL (Figure 3a) [58]. In addition to alkaline H_2_O_2_, a similar CL emission of QDs has also been observed in other ROS-related CL systems, including ONOO^−^ and HCO_3_^−^–H_2_O_2_ systems [25,59]. Similar to semiconductor QDs, direct CL of fluorescent CDs has also been reported, which can be regulated by regulating the surface-state luminescence of the CDs [20,60,61]. 

The fluorescent substance can act as an energy acceptor to form an excited state emitter when the energy of the active intermediate in the CL system matches the energy level of the fluorescent substance, thereby producing CL emission with a maximum emission wavelength consistent with the wavelength of its fluorescence. Since semiconductor QDs were reported as energy receptors in the luminol–H_2_O_2_ CL system in 2006, fluorescent nanomaterials show excellent potential as energy receptors in various CL systems [62,63,64,65,66]. CL probes or detection platforms constructed based on chemiluminescence resonance energy transfer (CRET) not only have higher sensitivity, but also possess a red-shifted emission wavelength, which would render them attractive candidates in bioanalysis. In order to reduce the CRET distance, Lu’s group created QD-LDH nanocomposites by organizing TGA-capped CdTe QDs in bilayer bunches on the outside of organo-modified LDH (Figure 3b), which greatly boosted the CL of the luminol–H_2_O_2_ system [62]. Lv’s group prepared oleic-acid-capped black phosphorus QDs (OA-BP QDs), which enhanced the ultra-weak CL of HSO_3_^−^–H_2_O_2_ and ClO^−^–H_2_O_2_ systems [66,67]. In these systems, OA-BP QDs catalyzed the triggering of the production of ^1^O_2_ and acted as energy acceptors to further strengthen the CL emission.

Similarly, fluorescent CDs and MNCs have shown potential as luminescence candidates due to their good biocompatibility, tunable emission wavelength and high photostability [68,69]. Recently, Shen et al. have designed a controlled nitrogen-doping route to prepare CDs with high-brightness CL by taking advantage of the energy level alignment between CDs and the high-energy intermediates generated in the CPPO–H_2_O_2_ system [27]. You et al. used BSA-stabilized AuNCs as energy acceptors to construct a CRET system, where TCPO–H_2_O_2_ CL reactions were used as energy donors [69]. In addition, some semiconducting polymer nanoparticles (SPN) and nanocrystals were also well-developed for CL emitters [70,71]. For instance, Lu et al. reported ultrathin Mn oxide [MnOx] nanosheet semiconducting polymer nanoparticles [SPNs], where the MnOx-generated ^1^O_2_ reacts with the thiophene units in the SPN, thereby exciting the SPN to emit NIR CL [70]. 

### 2.3. As Carriers of CL Reagents

**Figure 4:**
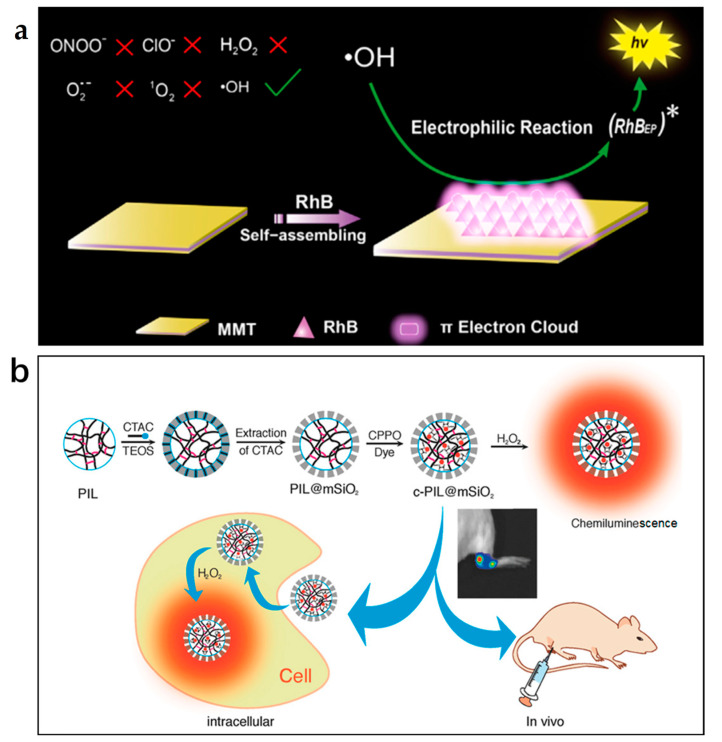
(**a**) LDH as a carrier of RhB for the CL enhancement. Reprinted with permission from ref. [73]. Copyright 2016 Elsevier. (**b**) Core–shell PIL@mSiO_2_ as a carrier of CPPO and dyes molecules. Reprinted with permission from ref. [77]. Copyright 2018 John Wiley and Sons.

Because of the substantial specific surface area and simplicity of modification of nanomaterials, they have been commonly used as carriers for CL reagents or enzymes, such as layered nanomaterials (e.g., LDH and montmorillonite), MOFs, etc. Yu et al. created an extremely sensitive CL probe by loading luminol into chromium(III) terephthalate MIL-101, which was deployed to detect H_2_O_2_ in aqueous solutions [72]. Lu’s group developed a serious of highly sensitive CL platforms using LDH and montmorillonite (MMT) as carriers of negatively/positively charged CL reagents [62,73]. Positively charged LDH can be used for the loading of anionic reagents such as luminol anions, and negatively charged MMT is an ideal carrier for the cationic reagents such as rhodamine B (RhB) (Figure 4a) [73]. The luminescence efficiency can be greatly improved because the lamellar structure of LDH and MMT with ordered charges can prevent the unordered stack of luminescent reagents on the surface of LDH and MMT layers. 

In addition to loading fluorescent dyes and luminescent reagents, nanomaterials have also been developed for loading enzymes. Luo et al. created a poly(ethylene-co-polyvinyl alcohol) (PVA-co-PE) nanofiber membrane with plenty of functional hydroxyl groups on its exterior for loading horseradish peroxidase (HRR), which displayed great activity, reusability, and sensitivity while catalyzing the luminol–H_2_O_2_ reaction [74]. Bagheri et al. synthesized LDH nanocomposites loaded with ZIF-8 (LDH@ZIF-8), which exhibited highly peroxidase-like activity and considerably increased the CL of the rhodamine B-H_2_O_2_ system [39]. 

In the peroxyoxalate (PO) CL system, the hydrophobic and unstable property of the CL reagents under aqueous conditions greatly hampered its use in biological systems. Therefore, various nanomaterial carriers such as micelles and nanocomposites were developed for loading POCL regents [75,76]. Zhou et al. prepared a core–shell structured nanocontainer (PIL@mSiO_2_) consisting of a poly-(ionic liquid) nanoparticle (PIL) core and a hydrophilic mesoporous silica shell to load CPPO and dye molecules [77]. The hydrophilic mesoporous silica shell not only provided the nanoprobe with chemical stability and hydrophilicity, but also permitted quick access of H_2_O_2_ to the CPPO in the hydrophobic PIL core (Figure 4b). The strategy of using PIL cores as hydrophobic nanocontainers for POCL reagents increased the mass transfer dynamics of micro-environmental H_2_O_2_ to POCL reagents. Additionally, the enormous pore capacity of mesoporous silica nanoparticles (MSNs) also allows for good encapsulation of large polymers to prevent environmental degradation of the polymer chains. The functionalization of external surfaces of MSNs permitted directed delivery and has been widely used in drug delivery systems. For instance, Li et al. employed MSNs modified via in situ polymerization with the conjugated polymer PPV to bind to the polymer poly[(9,9-di(2-ethylhexyl)-9H-fluorene-2,7-vinylene)-co-(1-methoxy-4-(2-ethylhexyloxy)-2,5-phenylenevinylene] (PFV-co-MEHPV), which could be oxidized by ClO^−^ to generate CL [78]. 

## 3. Nanomaterial-Based CL Probes for Biosensing and Bioimaging of ROS

### 3.1. H_2_O_2_

The absence of auto-fluorescence interference and phototoxicity of high-energy excitation light, as well as high signal-to-noise ratio and minimal perturbation, make the CL method advantageous for in vivo H_2_O_2_ sensing [79]. The luminol–H_2_O_2_ CL system generated widespread interest in the biosensing of H_2_O_2_. Horseradish peroxidase (HRR), a common catalyst used in the luminol–H_2_O_2_ system, is easily denatured as a natural enzyme under strong acid, base and high-temperature conditions. Therefore, the construction of efficient HRP immobilization systems to improve its reusability has become a hot research topic. Luo et al. prepared a PVA-co-PE nanofiber H_2_O_2_ sensor using the luminol–H_2_O_2_ CL system [74]. The PVA-co-PE nanofiber was modified by cyanuric chloride and 1,3-propanediamine before being biotinylated and then successfully immobilized with HRR. The HRP-immobilized PVA-co-PE nanofiber membrane demonstrated great activity, sensitivity, and reusability for the CL system of luminol–H_2_O_2_, which has potential for biological and medical applications. 

**Figure 5:**
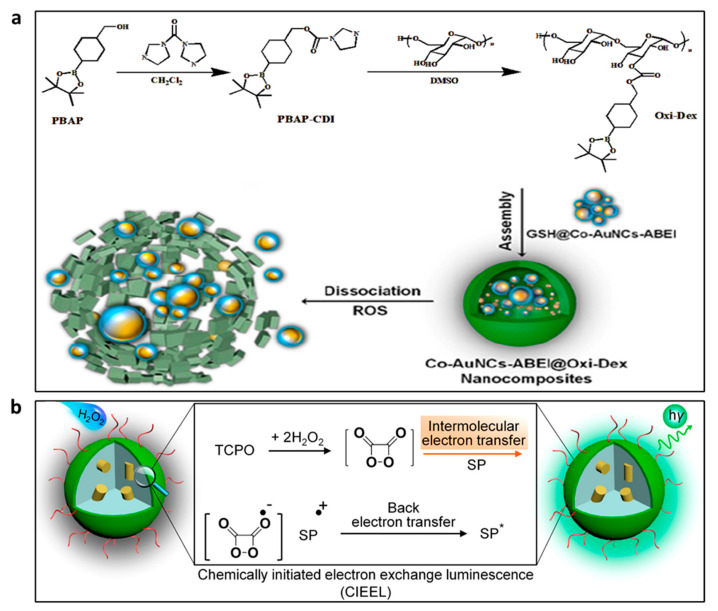
(**a**) The ABEI-functionalized Co-Au bimetallic cluster for H_2_O_2_ detection. Reprinted with permission from ref. [24]. Copyright 2020 American Chemical Society. (**b**) The diagram of the mechanism of SPNs for H_2_O_2_ imaging. Reprinted with permission from ref. [79]. Copyright 2016 American Chemical Society.

In recent years, noble-metal NCs including AuNCs, CuNCs and AgNCs have become more prevalent as catalysts than traditional ones such metal ions and enzymes. For example, Sheng et al. synthesized BSA-capped AgNCs to catalyze the luminol–H_2_O_2_ CL reaction for the detection of H_2_O_2_ and uric acid in human serum [35]. BSA-AgNCs were found to have a high affinity for H_2_O_2_ to facilitate the production of •OH. Created by the reaction between O_2_^•−^ and •OH, ^1^O_2_ directly oxidized luminol to yield CL. The luminol–H_2_O_2_-AgNC system was employed to detect uric acid in biological samples, and positive results were achieved. Some bimetallic clusters have also been reported for their catalytic role in H_2_O_2_ detection. Gao et al. reported a ROS stimulation-responsive CL nanocomposite for the extremely sensitive and selective determination of H_2_O_2_ in human serum samples [24]. The Co-Au bimetallic cluster with ABEI functionality was encapsulated in a biopolymer with boronic ester modifications to create the Co–AuNCs–ABEI@Oxi-Dex (Figure 5a). The modification of biocompatible polymers with boronic ester, sulfide and other substances has shown great potential for use as an ROS-responsive polymer material. With a linear range of 50 pM to 0.1 μM and a limit of detection of 35.8 pM, the CL sensor is highly sensitive, reagent-free and nonenzymatic, making it suitable for use in direct H_2_O_2_ sensing. 

Luminol is a frequently used CL reagent, but its poor aqueous solubility hinders its application in bioimaging. To overcome this problem, various luminol derivatives have been investigated to improve the aqueous solubility and increase the CL intensity. Zhang et al. developed the luminol analog L012 CL probe for in vivo imaging of H_2_O_2_ [80]. Furthermore, the CL chitosan hydrogel L012-Cs-Co^2+^ was created to extend the emission period and intensity through a slow-diffusion-controlled heterogeneous catalytic mechanism in order to improve analytical accuracy due to the flash-type photoemission of L012.

Compared with luminol CL systems, POCL systems have a longer emission time that is better suited for H_2_O_2_ imaging. However, it cannot be directly used in aqueous environments due to the limitation of water solubility and stability of peroxalate, which can be overcome by integrating POCL reagents in nanoparticles. Semiconductor polymer nanoparticles (SPNs) not only have high brightness and excellent photostability, but are also inert to ROS and can therefore be used as versatile nanoplatforms for the development of in vivo mouse ROS imaging probes [81]. Zhen et al. carried out energy level modulation by combining polyfluorene-based SPs with various molecular orbitals with peroxalate luminous substrates (TCPO), which is crucial for encouraging intermolecular electron transfer and CL augmentation during H_2_O_2_-driven luminescence (Figure 5b) [79]. The H_2_O_2_-activated luminescence of SPN was found to be primarily governed by the energy interval between the HOMO of SP and the LUMO of the high-energy intermediate dioxetanedione. The CL quantum yield (QY) and LOD of the optimized SPN were significantly better than those of the previous probes. Furthermore, the SPN could be co-doped with 2,3-naphthalocyanine dyes into nanoparticles to form a CRET to produce NIR luminescence (775 nm), which has been effective in imaging H_2_O_2_ at extremely low levels in living mice.

In addition, an enhanced NIR POCL signal can be obtained by a ‘nanophotonic energy relay’ approach [82], which did not require an emitter structure to meet the energy-matching requirement. The aggregation-induced emission (AIE)-active POCL nanoparticle was constructed by dense nano-integration of multiple molecules, including low-bandgap conjugated polymers with AIE effects as NIR emitters, peroxalate as a chemical fuel in response to inflammatory H_2_O_2_, and energy-gap-bridging photonic molecules. The energy-gap-bridging photonic molecules have been shown to efficiently accept energy from peroxalate chemically excited intermediates and relay it to the NIR emitter. The ‘nanophotonic energy relay’ method did enhance the H_2_O_2_-responsive NIR signal of the nanoparticle for sensitive imaging of deep inflammation. A CD-based CL probe for H_2_O_2_ in vitro and in vivo imaging was reported as a means of enhancing the chemical stability of CL emitters in highly oxidized ROS [83]. The probe (P-CDs) was prepared by nano-integration of near-infrared CDs and peroxalate CPPO, which exhibit significant advantages for H_2_O_2_ imaging, such as emission wavelength of the CDs in the NIR region and modulation of the energy level to shorten their energy gap with the intermediate-1,2-dioxetanedione, which facilitates the CL QY of the CDs (9.98 × 10^−3^ einstein mol^−1^, with fluorescein as the reference). 

**Figure 6:**
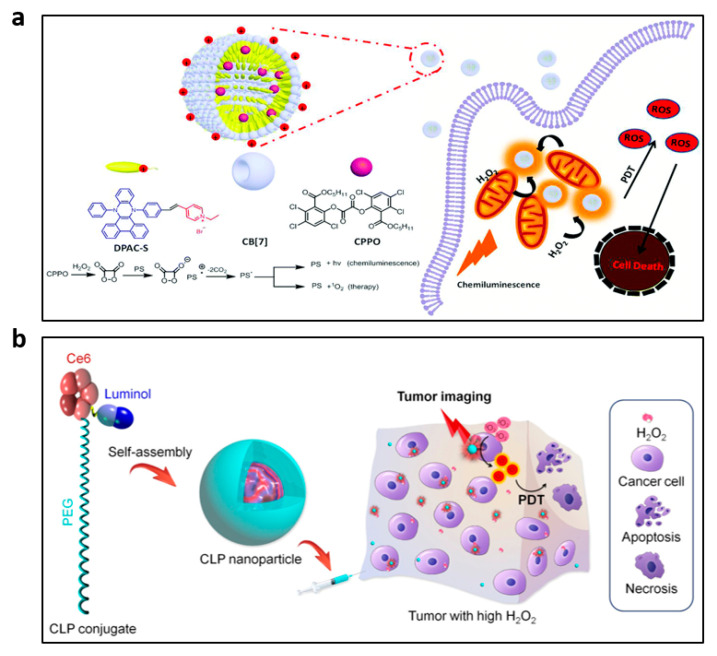
(**a**) Composition of the DPAC-S@CB[7]@CPPO and illustrations for PDT and cellular imaging. Reprinted with permission from ref. [85]. Copyright 2020 Royal Society of Chemistry. (**b**) Diagrammatic depiction of CLP-nanoparticle-mediated in situ PDT and luminescence imaging of cancers. Reprinted with permission from ref. [86]. Copyright 2020 American Chemical Society.

CL, a light source independent of tissue penetration depth, can effectively address the drawback of photodynamic therapy (PDT) brought on by external excitation light sources. To date, the absence of sensitive luminous chemicals has restricted PDT and luminescence imaging of cancers with high H_2_O_2_ levels. Thus, in recent years, different nanoprobes have been reported for H_2_O_2_ bioimaging and PDT of tumors. Mao et al. designed C-TBD nanoparticles (C-TBD NPs) as selective H_2_O_2_ probes by encapsulating the AIE-induced FR/NIR-emission photosensitizer TBD and CPPO into amphipathic pluronic F-127 in the presence of soybean oil [84]. The C-TBD NPs produced effective ^1^O_2_ to inhibit tumor growth in the presence of H_2_O_2_, and they have been employed for specific CL-excited in vivo tumor precision tracking and treatment. Chen et al. designed the DPAC-S@CB[7]@CPPO nanoparticles, which are used for mitochondria-target H_2_O_2_ imaging and in situ PDT, by combining the photosensitizer molecules 4,40-(dibenzo[a,c]phenazine-9,14-diyl)pyridinium bromide (DPAC-S), cucurbit[7]uril (CB[7]) and CPPO [85]. To produce DPAC-S@CB[7]@CPPO water soluble in aqueous solution, the hydrophobic molecule CPPO is co-assembled into nanoparticles. As a result, H_2_O_2_ can diffuse quickly into the assembly and react with CPPO. The nanoparticles are positively charged thanks to DPAC-S, which enables them to target mitochondria. CPPO interacts with H_2_O_2_ released from mitochondria and generates energy that is easily absorbed by nearby DPAC-S molecules efficiently to generate fluorescence emission. At the same time, the photosensitizer DPAC-S molecules can rapidly produce ^1^O_2_ in situ to kill cancer cells (Figure 6a). Similarly, the CL light from luminol–H_2_O_2_ system has also been used as the light source of PDT [86]. The amphiphilic conjugate of chlorin e6 (Ce6) concurrently conjugated with luminol and poly(ethyleneglycol) (CLP) yielded nanoparticles with a core–shell nanostructure. Due to the luminol unit and the excitation of Ce6 by CRET, CLP nanoparticles that are activated by H_2_O_2_ allow for in vitro and in vivo imaging of tumors with highly expressed H_2_O_2_. Moreover, the excited Ce6 can generate ^1^O_2_ and thus show anticancer activity for the in situ PDT of tumors (Figure 6b).

### 3.2. •OH

•OH radicals are extremely reactive and oxidative in many biological processes, such as the direct oxidation of biological macromolecules, for example, lipids, proteins and nucleic acids [73]. To fully understand the biological role of •OH in both biological and pathological events, it is crucial to develop sensitive and specific probes for •OH in biological systems [87].

**Figure 7:**
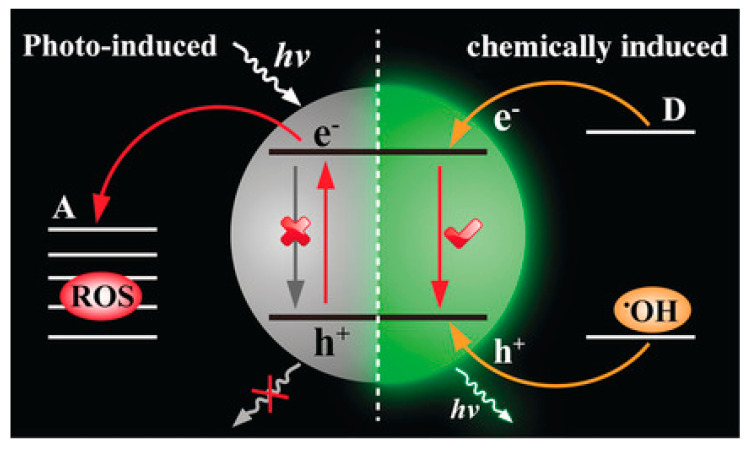
The QD-based CL probe for the specific detection of •OH. Reprinted with permission from ref. [26]. Copyright 2016 John Wiley and Sons.

To develop optical sensing systems for various cell-imaging methods, various functionalized semiconductor QDs have been developed for the purpose of identifying •OH. Among all ROS, •OH has the highest redox potential; thus, it can inject holes into QDs to form QDs^•−^. If there are electron donors in the system that can inject electrons into QDs to generate QD^•+^, CL emission appears through electron transfer compounding. Based on this, the Lu group created a QD-based turn-on luminous probe utilizing citrate as electron donors for the precise detection of •OH and enabled real-time monitoring of endogenous release of •OH in living cells (Figure 7) [26]. For the CL detection of •OH, in addition to the QD-based CL reaction, rhodamine-based CL systems have also been employed [73]. Cationic clay MMT which has negatively charged layers was used to support cationic rhodamine B, thereby preventing the disordered aggregation of rhodamine dyes. The strong electrophilicity of •OH allowed it to electrophilically attack rhodamine B (RhB), which is H-type aggregated on the surface of the MMT, triggering strong CL emission. The ability of the CL system to detect •OH in freshly drawn mouse plasma samples has been demonstrated.

### 3.3. O_2_^•−^

One of the main ROS, O_2_^•−^ radicals also serve as a source for many other free radicals produced inside of cells. Therefore, the high sensitivity and specificity of O_2_^•−^ detection in biological systems have attracted a lot of attention. Numerous nanoprobes for non-invasive in vivo CL imaging of O_2_^•−^ have become appealing options recently.

**Figure 8:**
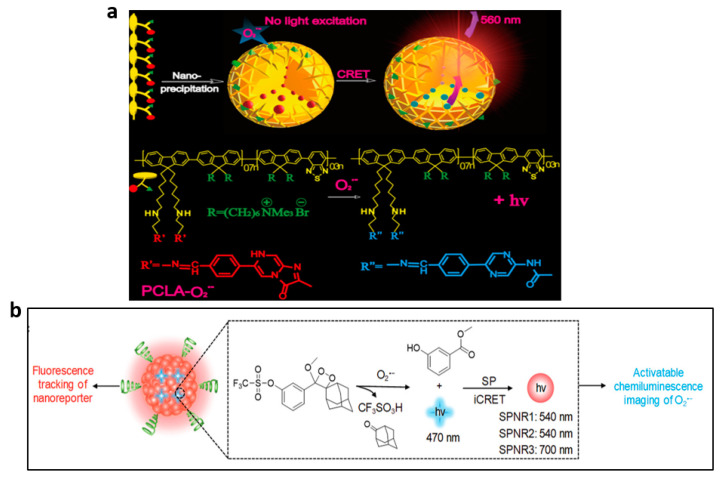
(**a**) Structure of the PCLA-O_2_^•−^ probe. Reprinted with permission from ref. [88]. Copyright 2016 American Chemical Society. (**b**) Description of the O_2_^•−^-activated chemiluminescence mechanism of SPNRs. Reprinted with permission from ref. [89]. Copyright 2019 John Wiley and Sons.

Li and associates developed a novel polymer nanoprobe based on CRET known as PCLA-O_2_^•−^ for monitoring O_2_^•−^ in mice in real-time [88]. The PCLA-O_2_^•−^ probe was made up of two components joined by a covalent bond: the imidazopyrazinone moiety (CLA), which served as the energy donor and O_2_^•−^ recognition unit, and the CPs (PFBT), which served as the signal-amplification matrix and the energy acceptor (Figure 8a). The PCLA-O_2_^•−^ polymer chains covalently linked with multiple CLAs folded in aqueous media to form dense spherical nanoparticles. Due to its extremely high sensitivity and specificity, significantly prolonged luminescence time and excellent biocompatibility, the PCLA-O_2_^•−^ probe could serve as a viable alternative method for in vivo imaging of ultralow levels of O_2_^•−^. It has been successfully used for real-time imaging of O_2_^•−^ in mice.

SPN-based CL imaging has been explored for the detection of ROS in animal models of malignancies, neuroinflammation and peritonitis, among others [79,84]. SPNs doped with peroxalate compounds have been shown to emit light by a chemical reaction, thus avoiding background noise and having the potential to image deep tissue in vivo with a relatively high signal-to-background ratio. Very recently, Cui et al. developed semiconductor polymer nano-reporters (SPNRs) that were activated by O_2_^•−^ to produce CL, which can be successfully used to image immune-activation in vivo [89]. SPNRs consisted of an SP and a caged CL phenoxy-dioxetane substrate responsive to O_2_^•−^, which acted as CL acceptors and donors, respectively, to produce CL based on a CRET mechanism (Figure 8b). Notably, the proposed SPNRs demonstrated CL at NIR region (*λ*_max_ = 700 nm) and are therefore appropriate for in vivo imaging of cancer immunotherapy and have the potential to enable high-throughput immunotherapeutic drug screening.

### 3.4. O_2_

^1^O_2_ plays a crucial role in many physiological processes that occur within living things, such as the activation of gene expression, the maintenance of immune system sterility and cell-signaling cascades [90,91,92]. Photosensitizers generate ^1^O_2_ by sensitizing surrounding molecular oxygen when irradiated by light, which can kill cancer cells and be useful in tumor photodynamic therapy (PDT) [93]. However, in vivo monitoring of ^1^O_2_ is generally challenging because of its short-lived state and extremely high reactivity.

CL has the distinct advantages of effectively avoiding photobleaching, light-scattering and autofluorescence of FL probes and providing a remarkably high signal-to-noise ratio [94]. It should be noted that CL also effectively prohibits the possible phototoxicity of the FL probe caused by the excitation light [95]. Many prior studies have been published exploring the CL detection of ^1^O_2_, but many problems remain to be solved, such as the low solubility of emission intermediate in water, extremely short half-life and the quenching effect of water molecules. Thus, the exploration of the role and mechanism of ^1^O_2_ action in biological and medical processes is greatly aided by the development of very effective CL probes for real-time sensing of ^1^O_2_.

**Figure 9:**
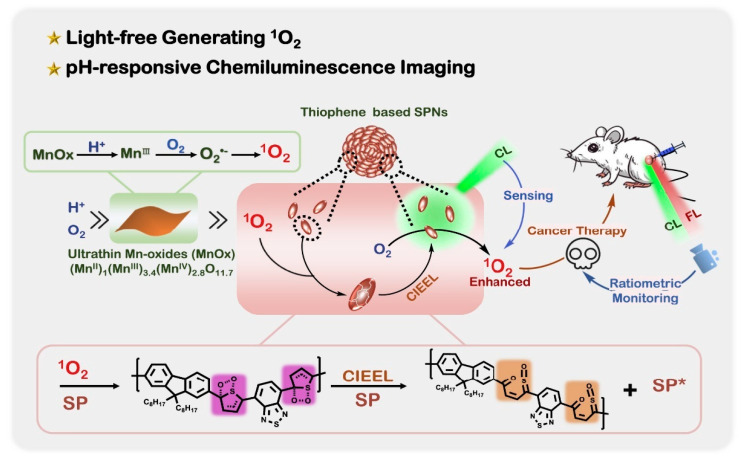
Schematic diagram of pH-responsive ^1^O_2_ production and CL imaging monitoring cancer treatment. Reprinted with permission from ref. [70]. Copyright 2020 Elsevier.

SPN, as a new type of optical reagent, has the advantages of adjustable optical properties, a large absorption coefficient and high biocompatibility [96]. It has been widely used as a nanoplatform for biomedical applications [97]. Lu et al. reported a chemodynamic and pH-responsive CL system, which is actually composed of ultrathin Mn oxide (MnOx) nanosheets and SPNs [70]. It is important to highlight the fact that ^1^O_2_ produced by MnOx can replace light irradiation, particularly by energizing thiophene-based SPN for NIR CL imaging and by increasing ^1^O_2_ production for chemodynamic therapy (Figure 9). In addition, CL/FL ratio imaging calibrated the output of ^1^O_2_ to more accurately monitor the chemodynamic process of treatment in situ. The therapeutic effects of the MnOx-SPN system were excellent both in vivo and in vitro.

^1^O_2_ formation in the human body can be simply divided into radiation-induced and nonradiation-induced. Because of its µs-level lifetime, radiation-induced cutaneous ^1^O_2_ is not sufficient to diffuse to other tissues, and nonradiation-induced ^1^O_2_ is of high significance in ^1^O_2_-related pathological effects and organ immune processes. Therefore, in vivo monitoring of nonradiation-induced ^1^O_2_ in real-time is among the most demanding tasks. Previously, a number of adamantylidene-based in vitro CL probes were developed that formed Schaap’s dioxetane via the reaction of alkene with ^1^O_2_, most of which were selective for ^1^O_2_, but their application in living specimens was probably limited by their hydrophobicity and poor CL quantum yield. There are still challenges with CL techniques for monitoring lower levels of ^1^O_2_ in ‘dark’ biological processes. Zhang et al. explored a new CL nanosensor (NTPE-PH) capable of responding specifically and selectively to ^1^O_2_ down to nM levels, providing a noninvasive method for characterizing ultra-trace nonradiation-induced ^1^O_2_ in whole animals [98]. The sensor possessed an ultra-high concentration of CL units prepared by the aggregation of synthetic tetraphenylethylene (TPE)-phthalhydrazid (PH) (TPE-PH) into high-fluorescence nanoparticles (NTPE-PH) in aqueous solution. The entire NTPE-PH is excited when the CL fraction in the NTPE-PH burns, which results in the production of an amplified CL. Through an intramolecular energy transfer mechanism, the NTPE-PH sensor promotes its remarkably high-energy transfer and ensures an aggregation-induced emission characteristic, producing a bright CL that is selectively sensitive to nM-level ^1^O_2_. Such sensors could be a practical tool for monitoring changes in ^1^O_2_ in immune responses and pathological processes corresponding to different stimuli and for exploring the biological role of ^1^O_2_.

### 3.5. ONOO^−^

ONOO^−^ can be obtained by the combination of reactive nitrogen radicals •NO and O_2_^•−^ radicals in biological systems. In addition, the •NO_2_ radical reacts with the •OH radical to produce its conjugate acid, ONOOH. The short-life ONOO^−^ is readily converted to other reactive secondary radicals such as •OH, •NO_2_ and •CO_3_^−^, which can cause reactions with biological macromolecules including lipids, nucleic acids and proteins, ultimately leading to immune responses, inflammation, cancer and other serious diseases [99]. To date, the biological mechanisms of ONOO^−^ involvement in the above-mentioned pathological processes have not been fully elucidated. Thus, the development of real-time, non-invasive, highly selective and sensitive approaches is essential for the identification of ONOO^−^ in vivo.

Luminol and its derivatives have been used for the detection of ONOO^−^, but with poor selectivity [100]. Several inorganic probes based on CDs, CdTe nanocrystals and organic molecular probes based on 1,2-dioxetane derivatives have been used for the CL detection of ONOO^−^ in solution or in living cells, but their use within in vivo CL imaging analysis is limited by emission wavelengths generally below 600 nm [101,102]. Thus, the development of CL probes with extended emission wavelengths is urgently needed.

For the luminol–H_2_O_2_-HRR CL system, fluorescent dyes were frequently used as CL energy receptors. For instance, it is possible for oxyanthracene fluorescent dyes to absorb energy in the excited state at 425 nm and then reemit it as brighter light at even longer wavelengths, usually 510–520 nm [103]. However, finding efficient methods to boost CRET efficiency remains a significant challenge because high fluorescent dye concentrations could frequently result in uncontrollable quenching effects brought on by aggregation. Wang et al. developed a CL probe for the specific detection of ONOO^−^ based on the strict CRET between an excited ROS donor (ONOOH*) and a fluorescent dye acceptor [28]. To reduce the quenching effect of aggregation brought on by high concentrations of fluorescent dye, they designed an ordered arrangement of fluorescent dye structures (calcein@SDS) by incorporating trace amounts of calcein molecules into a bilayer bundle of sodium dodecyl sulfate (SDS) exterior to the LDH. The method has been deployed successfully to identify ONOO^−^ in plasma samples of cancer mice, and it has shown a lot of promise for real-time tracking of intracellular ROS signals.

**Figure 10:**
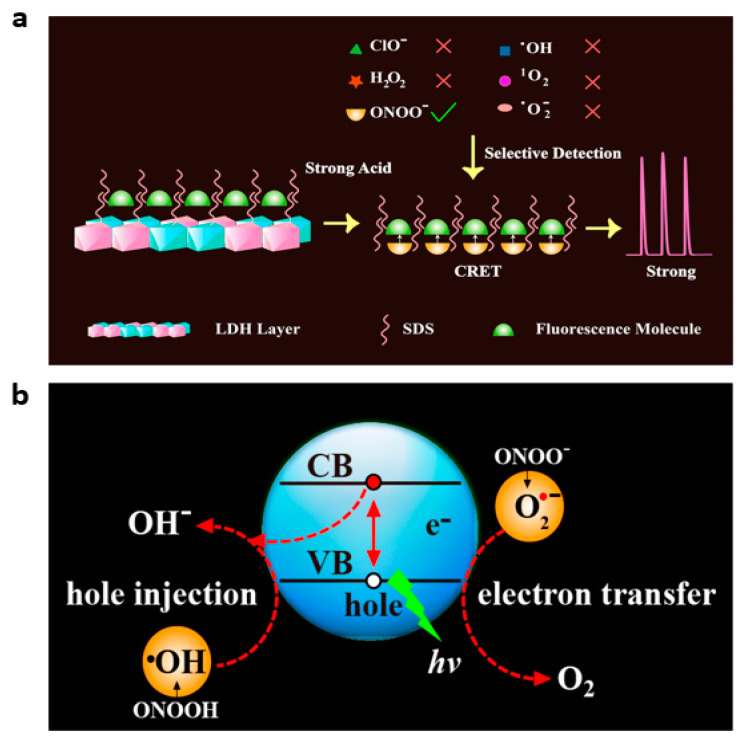
(**a**) The CL characteristics of calcein@SDS. Reprinted with permission from ref. [28]. Copyright 2015 American Chemical Society. (**b**) Diagrammatic representation of the QD-ONOO^−^ system’s CL emission process. Reprinted with permission from ref. [25]. Copyright 2016 American Chemical Society.

The CL-based ONOO^−^-specific detection was usually challenged by cross-interference from other ROS [104]. QDs are known to react with oxidation/reduction radicals very quickly, introducing holes or electrons to generate oxidized and reduced QDs, which then emit bright light through a process called electron transfer annihilation [105]. The use of interactions between QDs and ROS to enable the detection of ROS has attracted research interest. Lu’s group reported a QD-based CL system for the incredibly selective determination of ONOO^−^ in live cells [25]. The mechanism of the system mentioned above is as follows (Figure 10b). Oxidized QDs (QDs^•+^) are created when oxidizing radicals •OH from the breakdown of ONOO^-^ insert holes into the valence band (VB) of TGA-CdTe QDs. After that, QDs^•+^ and O_2_^•−^ (also from ONOO^−^) undergo electron transfer annihilation, creating excited QDs that release light as they return to their ground state. The QD-based CL probe showed excellent selectivity for ONOO^−^ among all ROS. To further improve the sensitivity and biotoxicity, they constructed a CD-based CL probe by adjusting the surface-state luminescence of CDs and successfully applied it to identify endogenous ONOO^−^ in live cells [20].

CL imaging of ONOO^−^ in vivo requires longer wavelengths of emission light (preferably in the NIR). For CL imaging of endogenous ONOO^−^ in mice, Wang et al. created a new nanoprobe (NPs-PCP) by nanoprecipitating oxygen-embedded quinoidal pentacene (O-Pentacene) with a near-IR semiconductor polymer (PCPDTBT) [99]. The association of ONOO^−^ with O-Pentacene may result in the production of a light-emitting intermediate with high energy (carboxy O-Acene). To increase the luminescence wavelength to the NIR, the PCPDTBT was co-precipitated into the nanoprobe, which was capable of receiving the energy transferred from O-Pentacene via CRET. The nanoprobe presented excellent selectivity and ultra-high sensitivity for imaging ONOO^−^.

### 3.6. HClO/ClO^−^

HClO is formed in living organisms by the reaction of H_2_O_2_ with Cl^−^ catalyzed by a heme protease, MPO, and is localized primarily to macrophages/monocytes, neutrophils and microglia in the brain [106]. In biological fluids, the higher concentrations of Cl^−^ react with most of the H_2_O_2_ to form HClO. The generated HClO may also form •OH by reacting with O_2_^•−^ [107]. In animals or humans, HClO is also considered a crucial microbicidal agent for the duration of the immune response, for example, in relation to the digestion of bacteria swallowed by neutrophil phagosomes [108]. The overbalanced production of HClO during inflammation may contribute to the pathogenesis of several types of illnesses involving inflammatory bowel disease, atherosclerosis, myocardial infarction and even cancer, among others [109]. Rapidly and efficiently detecting HClO is of vital significance for biological studies associated with its biological functions, especially for the study of the concentration levels, metabolism, production and distribution of HClO in living organisms.

**Figure 11:**
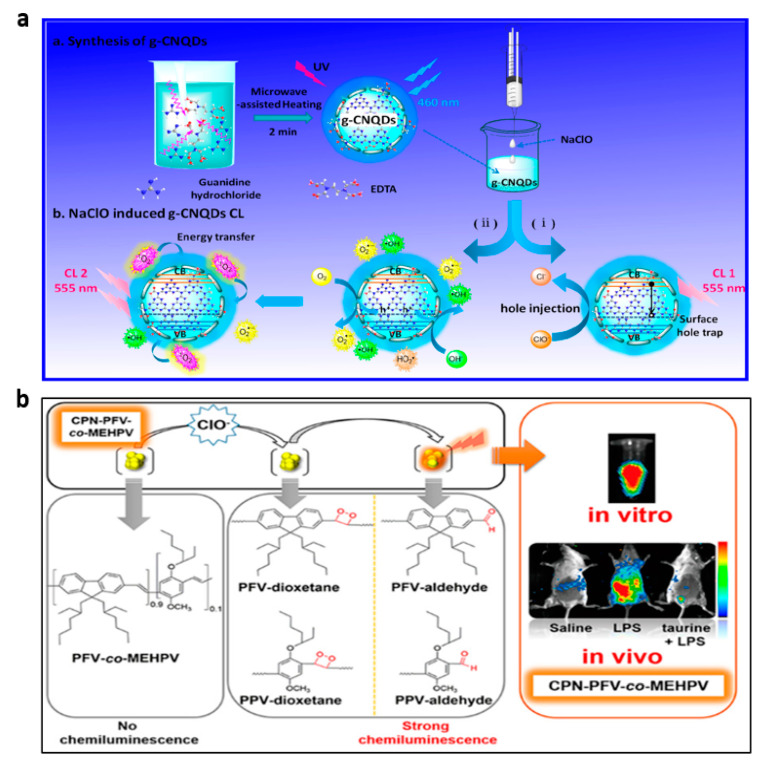
(**a**) Mechanism of CL in the g-CNQDs-NaClO system. Reprinted with permission from ref. [105]. Copyright 2014 American Chemical Society. (**b**) The CPN-PFV-co-MEHPV for endogenous ClO^−^ in situ imaging. Reprinted with permission from ref. [110]. Copyright 2018 American Chemical Society.

QD nanomaterials have good catalytic, optical and biocompatible properties, and their use for ClO^−^ detection has also attracted attention. It has been noted that some QDs, especially carbon nitride QDs and black phosphorus QDs, release CL when they come into interaction with ClO^−^ [67,105]. Black phosphorus QD (BP QD) exhibited excellent CL properties when H_2_O_2_ and ClO^−^ are present [67]. The CL mechanism of the BP QDs-H_2_O_2_-ClO^−^ system has been investigated as the reaction of ROS (•OH, ^1^O_2_, etc.) with BP QDs to generate excited phosphorus oxides (HPO), which emit an intense CL signal at approximately 530 nm. In the absence of H_2_O_2_, carbon nitride QDs (g-CNQDs) exhibited strong CL when injected with NaClO, resulting in the development of a new CL system for the direct detection of free chlorine in water [105]. The radiative recombination of holes and electrons induced by the oxidant in the g-CNQDs was identified as the most probable CL mechanism for the g-CNQDs-NaClO system (Figure 11a). Additionally, the g-CNQDs could receive energy from the ^1^O_2_ formed by the interaction of other ROS present in the system, such as •OH and O_2_^•−^, which would increase CL emission.

In addition to QDs, conjugated polymers (CPs) have also been reported to yield CL emission when directly oxidized by ClO^−^ [110]. Delocalized electronic structures in the conjugated backbone of CPs promotes the transmitting of excited-state energy with high efficiency within or between chains to energy receptor sites, increasing the receptor signal in the process. Thus, an easier approach using straightforward CL nanoprobes for imaging was made possible by the strategy of directing the oxidation of CPs through ROS to produce CL signals. Zhu et al. reported a CP-based CL nanoprobe (CPN-PFV-co-MEHPV) for ClO^−^ detection [110]. The CL mechanism of the CPN-PFV-co-MEHPV probe can be described as follows: ClO^−^ via π-π cycloaddition oxidized the vinylene bond (C=C) in the polyfluorene-vinylene (PFV)/polyphenylene-vinylene (PPV) derivatives leading to a high-energy intermediate, the dioxetane motif, which was spontaneously degraded and yielded CL. Such a CP-based CL system avoided the problem of CL donor leakage from the nanoparticles as it did not require the involvement of small-molecule CL donors at all. The CPN-PFV-co-MEHPV probe has been demonstrated to be non-cytotoxic, sensitive and selective toward ClO^−^ and has been successfully implemented for visualizing endogenous ClO^−^ in living animals (Figure 11b).

In addition, encapsulating CPs in nanoparticle form in aqueous solutions separates unstable dioxetane intermediates from solvent molecules, resulting in an increase in CL emissions. The inner and outer surfaces of MSN contain an extensive range of silanol groups that can be modified with diverse organic functional groups through electrostatic interaction or covalent bonding. MSN has also been developed to load CPs for in vitro and in vivo detection of ClO^−^ activity. Recently, Li et al. encapsulated CP (CP1) in polystyrene (PPV) pre-modified MSN, resulting in the construction of luminous PPV@MSN-CP1 nanoparticles which were employed for the detection of ClO^−^ in tumor imaging [78]. The mechanism of luminescence of the nanoparticles (PPV@MSN-CP1) is the oxidation of the vinylidene bond of CP1 by hypochlorite via π^2^-π^2^ cycloaddition, followed by the formation of a PPV-dioxetane intermediate to generate photons.

## 4. Conclusions

**Figure 12:**
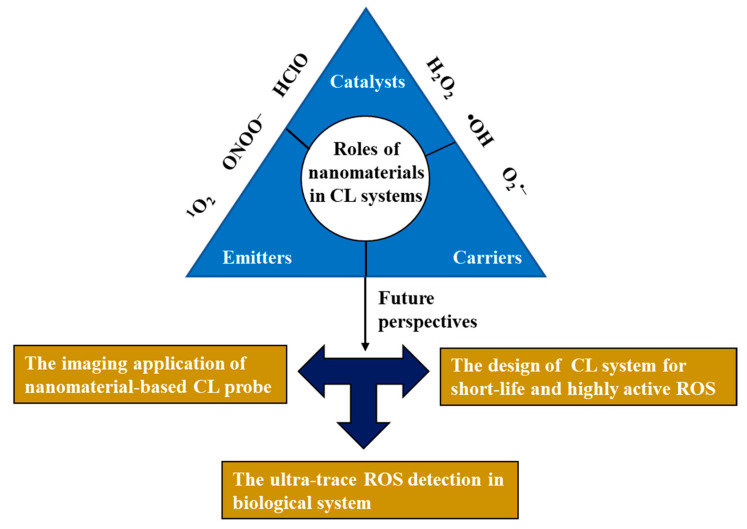
Schematic diagram of the principle of nanomaterial-enhanced CL and future perspective of nanomaterial-based CL probes for ROS detection.

ROS in biological systems have attracted wide attention due to their vital roles in maintaining normal physiological activities. The imbalance of ROS in organisms will cause damage to organisms and induce a variety of diseases. Therefore, it is very important to develop highly sensitive and selective platform for ROS quantitative analysis. The CL method has been widely used in bioanalysis, biosensing and imaging due to its high sensitivity, lack of background interference and simple instruments. In recent years, the application of nanomaterial-based CL systems in ROS sensing has been extensively studied. In this review, the principle of nanomaterial-enhanced CL, the construction of nanomaterial-based CL probes and their applications in ROS biosensing are reviewed (Figure 12).

Up to now, the main challenge in developing desirable CL probes for ROS lies in the selectivity and sensitivity in usage, and particularly in complex systems involving biological samples and living cells. The sensitive detection of most ROS is limited by their low levels and short lifetimes in biological systems. The incorporation of various nanomaterials such as QDs, MOF, LDH and noble-metal nanoparticles has created a lot of new possibilities for the application of various CL systems. By virtue of the biocompatibility, catalytic activity and adsorption capacity or optical properties of nanomaterials, various nanomaterial-based CL probes have given a valuable promotion to ROS sensing and imaging in living organisms. For example, improvements in CRET efficiency, the increase in emission wavelength to the near infrared region for enhanced tissue penetration, the enhancement of CL intensity to improve the sensitivity of ROSs detection, etc.

Notwithstanding the encouraging progress, a number of concerns remain. Firstly, there are only a few CL probes specifically designed for sensing ^1^O_2_, O_2_^•−^, ClO^−^ and ONOO^−^ in comparison to H_2_O_2_. More attention should be paid to these kinds of short-life and highly active ROS in the design and development of new CL reagents/systems. Secondly, the sensitivity and selectivity of existing CL probes still need to be improved. We should make more efforts for the detection of ultra-trace ROS in biological systems by making full use of the structure and properties of nanomaterials. Finally, the imaging application of nanomaterial-based CL in vivo remains greatly limited. Long-lasting and NIR-emissive CL systems should be a focal point to promote the application of CL imaging in vivo. In short, nanomaterial-based CL, which has a wide range of application prospects, still needs more in-depth study and exploration. This review is expected to provide a reference for further expanding the application of nanomaterial-based CL in ROS biosensing and imaging applications.

## Data Availability

Not applicable.
